# The DPP4 Inhibitor Linagliptin Protects from Experimental Diabetic Retinopathy

**DOI:** 10.1371/journal.pone.0167853

**Published:** 2016-12-12

**Authors:** Nadine Dietrich, Matthias Kolibabka, Stephanie Busch, Petra Bugert, Ulrike Kaiser, Jihong Lin, Thomas Fleming, Michael Morcos, Thomas Klein, Andrea Schlotterer, Hans-Peter Hammes

**Affiliations:** 1 5th Medical Department, Universitätsmedizin Mannheim, University of Heidelberg, Mannheim, Germany; 2 Department of Medicine I, University of Heidelberg, Heidelberg, Germany; 3 Department of CardioMetabolic Diseases Research, Boehringer Ingelheim Pharma, Biberach, Germany; Queen's University Belfast, UNITED KINGDOM

## Abstract

**Background/aims:**

Dipeptidyl peptidase 4 (DPP4) inhibitors improve glycemic control in type 2 diabetes, however, their influence on the retinal neurovascular unit remains unclear.

**Methods:**

Vasculo- and neuroprotective effects were assessed in experimental diabetic retinopathy and high glucose-cultivated *C*. *elegans*, respectively. In STZ-diabetic Wistar rats (diabetes duration of 24 weeks), DPP4 activity (fluorometric assay), GLP-1 (ELISA), methylglyoxal (LC-MS/MS), acellular capillaries and pericytes (quantitative retinal morphometry), SDF-1a and heme oxygenase-1 (ELISA), HMGB-1, Iba1 and Thy1.1 (immunohistochemistry), nuclei in the ganglion cell layer, GFAP (western blot), and IL-1beta, Icam1, Cxcr4, catalase and beta-actin (quantitative RT-PCR) were determined. In *C*. *elegans*, neuronal function was determined using worm tracking software.

**Results:**

Linagliptin decreased DPP4 activity by 77% and resulted in an 11.5-fold increase in active GLP-1. Blood glucose and HbA_1c_ were reduced by 13% and 14% and retinal methylglyoxal by 66%. The increase in acellular capillaries was diminished by 70% and linagliptin prevented the loss of pericytes and retinal ganglion cells. The rise in Iba-1 positive microglia was reduced by 73% with linagliptin. In addition, the increase in retinal Il1b expression was decreased by 65%. As a functional correlate, impairment of motility (body bending frequency) was significantly prevented in *C*. *elegans*.

**Conclusion:**

Our data suggest that linagliptin has a protective effect on the microvasculature of the diabetic retina, most likely due to a combination of neuroprotective and antioxidative effects of linagliptin on the neurovascular unit.

## Introduction

Diabetic retinopathy remains highly prevalent despite substantial progress in therapeutic approaches [1]. In endothelial cells and retinae of diabetic animals, hyperglycemia causes mitochondrial overproduction of reactive oxygen species (ROS) and subsequent formation of methylglyoxal (MG) as well as accumulation of MG-derived advanced glycation endproducts (AGEs) [2, 3]. Beyond the early glycemia-driven stages of diabetic retinopathy, the propagation of inflammatory mediators such as Interleukin 1-beta (Il1b), monocyte chemotactic protein-1 (MCP-1) and adhesion molecules are upregulated and can contribute to capillary damage [4].

Glucagon-like peptide 1 (GLP-1) improves glycemic control in type 2 diabetes by affecting glucose-stimulated insulin secretion, gastric emptying, and hepatic glucose production [5]. Moreover, both GLP-1 and inhibitors of dipeptidyl peptidase 4 (DPP4) have protective effects on the cardiovascular system by different mechanisms [6]. In addition, GLP-1 can block overproduction of ROS and downstream expression of pro-inflammatory effectors by endothelial cells during hyperglycemia [7]. In animal models of diabetes, GLP-1 also demonstrates antioxidative effects on the vasculature [8]. In these preclinical models, treatment with GLP-1 agonists leads to reduced apoptosis and increased cell survival [9]. Data from preclinical and clinical studies suggest that long-term GLP-1 treatment protects the macrovasculature in diabetes, evidenced by reduced inflammatory signaling in macrophages, improved plasma lipid profiles, and reduced blood pressure [10–12]. Intravitreal injection of GLP-1 transiently improves neuronal function and reduces glutamate toxicity in diabetic rats [13]. These observations suggest a benefit of GLP-1 on the retinal neurovascular unit.

GLP-1 is rapidly degraded by DPP4. However, DPP4 has multiple other substrates for cleavage, which might be relevant in the diabetic retina, such as stromal cell-derived factor-1 alpha (SDF-1a). SDF-1a is increased in proliferative diabetic retinopathy and promotes angiogenesis [14]. Animals overexpressing SDF-1a develop more neovascularizations in ischemic tissues [15]. It is unknown whether reduction of SDF-1a contributes to early vasoregression. On the other hand, degradation products of active GLP-1, such as GLP-1(9–37) and GLP-1(9–36) amide, are putative inhibitors of mitochondrial ROS overproduction [16, 17]. Thus, DPP4 inhibition might also reduce vascular protection provided by GLP-1 cleavage products. Taken together, these conflicting effects make it difficult to predict the net effect of DPP4 inhibition on diabetic microvascular damage.

In this study, we investigated the effect of the DPP4 inhibitor linagliptin on experimental diabetic retinopathy in the rat. Since glycemia is a risk factor of microvascular damage, we chose the streptozotocin(STZ)-induced diabetic rat as this model does not respond with glucose normalization upon DPP4 inhibition. *Caenorhabditis elegans* (*C*. *elegans*) was used as a surrogate model for evaluating the protective effect of linagliptin on neuronal function.

## Materials and Methods

### Rats

The use of animals in the study was in accordance with institutional guidelines and in compliance with the Association for Research in Vision and Ophthalmology (ARVO) statement. All animal work was approved by the local ethics committee (Regierungspräsidium, Karlsruhe, Germany). Male 6-week-old Wistar rats (Harlan Winkelmann, Borchen, Germany) were rendered diabetic by intravenous injection of STZ (35 mg/kg body weight; Roche Diagnostics GmbH, Mannheim, Germany). Rats with a blood glucose level above 14 mmol/L (250 mg/dL), one week after STZ injection, were considered diabetic (D, n = 22). Rats with a blood glucose level above 33 mmol/L (600 mg/dL) were administered two IE insulin glargin (Lantus®, Sanofi, Frankfurt) subcutaneously three times per week. Linagliptin (Boehringer Ingelheim, Germany) was administered to diabetic rats in food pellets at a concentration of 0.083 mg/kg (Harlan Winkelmann, Basel, Switzerland) from Week 1 to Week 24 (D+Lina, n = 22) after STZ treatment. Dose was selected to yield a clinically relevant plasma concentration of 82 ± 17 nM. Glucose levels and body weight were monitored. At the end of the study, hemoglobin A_1c_ (HbA_1c_) was determined using an In2It analyzer (Bio-Rad Laboratories, Munich, Germany). Age-matched male Wistar rats served as controls (N, n = 22). 24 weeks after diabetes induction, rats were sacrificed under deep anesthesia and eyes and plasma samples were collected. For anesthesia, 80 mg/kg body weight ketamine and 4 mg/kg body weight xylazon was administered intraperitoneally.

#### Plasma DPP4 activity and GLP-1

Activity of DPP4 was measured using a specific peptide substrate with a terminal coumarin derivative (H-Ala-Pro-7-amido-4-trifluoromethylcoumarin; Bachem, Bubendorf, Switzerland) allowing quantification in a fluorescence microplate reader (Perkin Elmer Wallac Victor™ 1420 Multilabel Counter, Waltham, MS, USA) upon cleavage by DPP4 [18]. Total GLP-1 (all endogenous isoforms) and active GLP-1 concentrations (GLP-1 (7–36) amide and GLP-1 (7–37)) were determined using a commercially available ELISA kit (K150JVC-1 and K150JWC-1, Meso Scale Discovery, Gaithersburg, MD, USA).

#### Determination of methylglyoxal

The synthesis and purification of methylglyoxal (MG) as well as the synthesis of the derivatizing agent and the standards for determination of MG were prepared according to published procedures [19]. MG concentration in retinal tissue was determined by derivatization with 1,2-diamino-4,5-dimethoxybenzene. The protein concentration of the tissue homogenate was determined using the Bradford assay (Bio-Rad Laboratories, München, Germany).

#### Retinal digest preparation and quantitative retinal morphometry

Quantitative retinal morphometry was performed on retinal digest preparations to evaluate numbers of acellular capillaries (AC/mm^2^ retinal area) and pericytes (PC/mm^2^ capillary area), according to published methods [20].

#### SDF-1a and Ho-1 ELISA

Frozen retinae were homogenized in 300 μl PBS for SDF-1a ELISA (MCX120, R&D systems, Wiesbaden, Germany) and in 5x extraction reagent for heme oxygenase-1 (Ho-1) ELISA (EKS-810A, Stressgen/Biomol, Hamburg, Germany). Protein concentration was measured by Bradford assay (Bio-Rad Laboratories, München, Germany). The lysates were used for the ELISA according to the manufacturer’s instructions.

#### Immunohistochemistry

Retinal paraffin-embedded sections or whole retinae were incubated at 4°C overnight with the following primary antibodies: rabbit anti-rat high mobility group box 1 protein (HMGB-1) (Upstate/Millipore, Schwalbach, Germany), rabbit anti-rat ionized calcium-binding factor adaptor molecule 1 (Iba1) (Wako Chemicals, Neuss, Germany), rabbit anti-rat DPP4 (Abcam, Cambridge, UK), mouse anti-rat thymocyte antigen-1.1 (Thy1.1) (AbD Serotec, Düsseldorf, Germany), and rabbit anti-rat GLP-1 receptor (GLP-1R) (Abcam, Cambridge, UK). Sections or whole retinae were incubated with the following secondary antibodies: swine anti-rabbit FITC (Dako Cytomation, Hamburg, Germany) for HMGB-1, DPP4 and GLP-1R, donkey anti-rabbit AF 555 (Life Technologies, Darmstadt, Germany) for Iba1, and rabbit anti-mouse TRITC (Dako Cytomation, Hamburg, Germany) for Thy1.1. The sections or whole retinae were covered with Vectashield mounting medium (Vector/Linaris, Dossenheim, Germany). Photos were taken using a confocal microscope (Leica, Wetzlar, Germany) and microglial cells positive for Iba1 were quantified per mm² in whole retinae.

#### Quantification of nuclei in the ganglion cell layer

For the quantification of the ganglion cells, nuclei were counted within the ganglion cell layer (GCL) using 3 μm periodic acid-Schiff (PAS) stained paraffin sections. To cover both central and peripheral retina, eight sections per eye were analyzed, comprising the entire circumferential segment of the retina. Nuclei were counted on a horizontal length of 180 μm (nuclei in GCL/180 μm retina) using quantitative image analysis (Olympus Opticals, Hamburg, Germany).

#### GFAP Western blot

The isolated retinae were homogenized in 120 μl 0.1% SDS lysis buffer and protein concentration was determined by Bradford assay (Bio-Rad Laboratories, München, Germany). Samples were separated in a 4–15% gradient Tris-HCl Gel (Bio-Rad Laboratories, München, Germany) and immunoblotted to a polyvinylidene difluoride membrane (Sigma-Aldrich, München, Germany). Non-specific binding was blocked by incubation with 5% non-fat dry milk in TBS, containing 0.1% Tween (Sigma-Aldrich, Darmstadt, Germany), followed by overnight incubation at 4°C with rabbit anti-rat glial fibrillary acidic protein (GFAP) (Dako Cytomation, Hamburg, Germany) or rabbit anti-rat beta Tubulin (Abcam, Cambridge, UK) antibodies. For detection, an anti-rabbit horseradish-peroxidase antibody (Dako Cytomation, Hamburg, Germany) was used. Immunoreactive bands were visualized by incubation with chemiluminescence reagent (Perkin Elmer, Boston, MA, USA) and signals were detected with the Fusion SL (VWR, Darmstadt, Germany). Integrated densities were measured with ImageJ software [21].

#### Quantitative RT-PCR

Gene expression was assessed using quantitative RT-PCR. Retinal RNA was isolated and homogenized in Trizol reagent (Invitrogen, Karlsruhe, Germany). Reverse transcription of RNA was achieved using the QuantiTect Reverse Transcription kit (Qiagen GmbH, Hilden, Germany) and subjected to TaqMan^®^ analysis (Applied Biosystems, Weiterstadt, Germany). Gene expression was analyzed by the comparative delta-delta CT method using beta-actin as a housekeeping gene. The following primers and probes were purchased from Applied Biosystems (Weiterstadt, Germany):

IL1b (NM_031512.2, Rn00580432_m1), intracellular adhesion molecule-1 (Icam1) (NM_012967.1, Rn00564227_m1), C-X-C chemokine receptor type 4 (Cxcr4) (NM_022205.3, Rn01483207_m1), catalase (Cat) (NM_012520.1, Rn00560930_m1), GLP-1R (NM_012728.1, Rn00562406_m1) and beta-actin (NM_031144.3, Rn00667869_m1).

### Maintenance and experimental exposure of *C*. *elegans*

*C*. *elegans* were cultivated on nematode growth medium (NGM) agar on 60 mm Petri dishes and maintained at 20°C. Living *E*. *coli* bacteria (OP50) provided the food source. 100 μl of a standardized overnight culture was added to NGM plates using published protocols [22]. The wild type strain N2 was provided by the Caenorhabditis Genetics Center which is funded by National Institutes of Health Office of Research Infrastructure Programs (P40 OD010440).

To obtain age-synchronized nematodes, self-fertilizing hermaphrodites (3-days-old) were allowed to lay eggs. Hermaphrodites were subsequently removed to synchronize the eggs. After hatching and reaching adulthood, *C*. *elegans* were transferred to NGM agar plates containing 300 μg/ml 5-fluorodesoxyuridine (FUdR, Sigma-Aldrich, München, Germany) to prevent further hatching. These conditions were used as standard (N).

High glucose conditions in *C*. *elegans* were established after transferring nematodes to NGM-FUdR plates, by using 150 μl of a 400 mmol/L glucose solution daily, resulting in a whole-body concentration of 13.9 mmol/L (250 mg/dL) glucose in a *C*. *elegans* extract (D), mimicking clinical hyperglycemia, according to protocols described before [22, 23]. For additional treatment, linagliptin was added at a concentration of 13 μM to the high glucose solution (D+Lina). Evaluation of neuronal function was performed upon cultivation of animals for 12 days under N, D, or D+Lina conditions.

#### Analysis of neuronal function

High glucose induced neuronal damage was assessed by quantification of animal motility using established protocols [24]. In brief, single animals were maintained on NGM plates and recorded on video (Moticam 1000, Beyersdörfer GmbH, Mandelbachtal, Germany) at Day 12. For detailed analysis worm tracking software was used (WormTracker 4.0, Thomas Bornhaupt, Neustadt a. d. W., Germany) in order to calculate body bending frequency (n = 10 per group).

#### Statistical analysis

Data are presented as mean ± SD. Differences between groups were analyzed by ANOVA with the Bonferroni post-hoc method for multiple comparisons. Statistical analysis was performed using GraphPad Prism (GraphPad Software, San Diego, CA, USA). For all comparisons, a value of *P* < 0.05 was considered statistically significant.

## Results

**Fig 1:**
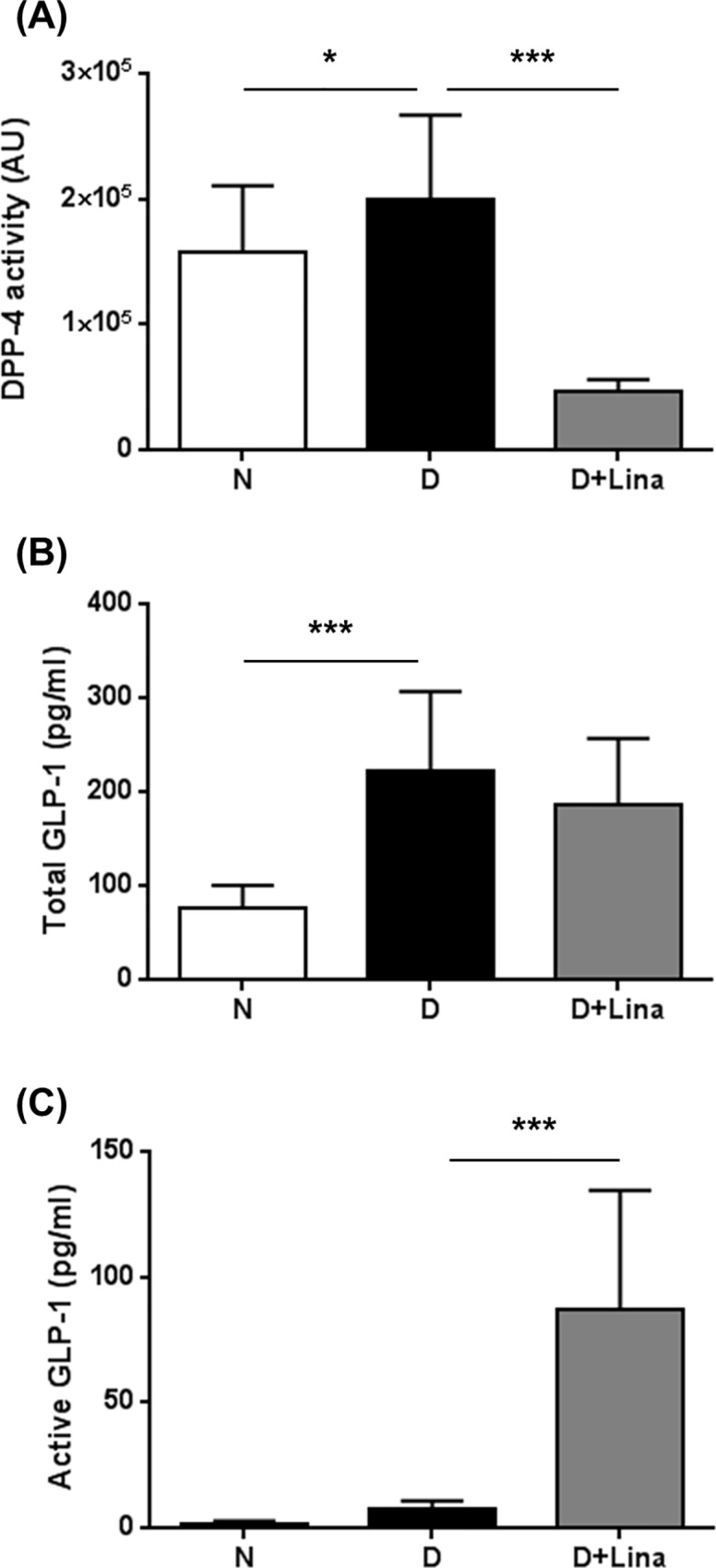
Pharmacological activity of linagliptin. (A) Plasma DPP4 activity was quantified by spectrophotometric monitoring in nondiabetic [N], diabetic [D] and linagliptin-treated diabetic animals [D+Lina], (B) total plasma GLP-1 and (C) active plasma GLP-1 were measured by ELISA. Data are expressed as mean ± SD. *P < 0.05, ***P < 0.001, (n = 22 for all parameters).

We evaluated the pharmacological activity of the DPP4 inhibitor linagliptin. Diabetic rats received the DPP4 inhibitor linagliptin within food pellets at a concentration of 0.083 mg/kg from Week 1 to Week 24 after STZ treatment. Diabetes led to a 26% increase in plasma DPP4 activity ([N] 1.6 ± 0.5 x 10^5^ AU vs [D] 2.0 ± 0.7 x 10^5^ AU; *P* < 0.05), whereas linagliptin reduced DPP4 activity by 77% (0.5 ± 0.1 x 10^5^ AU vs [D] *P* < 0.001) (Fig 1A). Under hyperglycemic conditions, total GLP-1 was elevated 2.9-fold compared with controls ([N] 77 ± 24 pg/ml vs [D] 221 ± 85 pg/ml; *P* < 0.001) and linagliptin did not influence the total content of GLP-1 peptides (Fig 1B).

Likewise, the concentration of active GLP-1 was increased by 4.6-fold ([N] 1.7 ± 1.0 pg/ml vs [D] 7.6 ± 3.1 pg/ml; P < 0.05). However, upon DPP4 inhibition, active GLP-1 was significantly increased by 11.5-fold ([D] 7 ± 3 pg/ml vs [D+Lina] 87 ± 47 pg/ml; *P* < 0.001) (Fig 1C).

**Fig 2:**
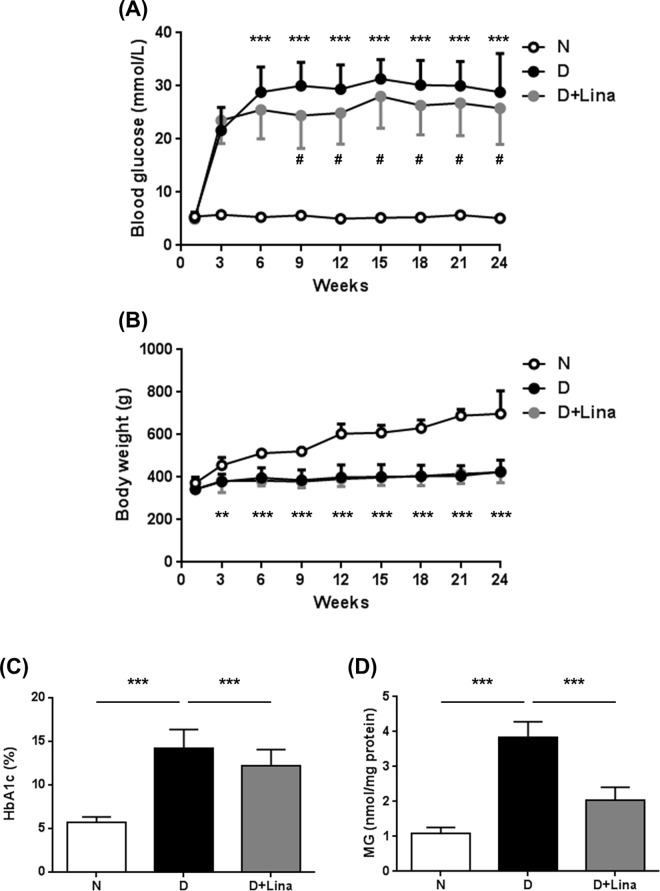
Metabolic effects of linagliptin. (A) Blood glucose (n = 22), (B) body weight (n = 22), (C) HbA_1c_ (measured by affinity chromatography, n = 22), and (D) retinal MG (measured by derivatization with 1,2-diamino-4,5-dimethoxybenzene using HPLC, n = 5) were determined. Data are expressed as mean ± SD. **P < 0.01, ***P < 0.001.

We assessed changes in metabolic parameters of diabetic animals upon linagliptin treatment. Diabetes induction resulted in persistent hyperglycemia over 24 weeks. Average glycemia was 5.3 ± 0.7 mmol/L in non-diabetic animals, and 30.0 ± 4.9 mmol/L in diabetic animals. Linagliptin had only a minor impact on glycemia in STZ diabetic animals, a model in which more than 90% of beta cells are destroyed. Diabetic animals receiving linagliptin had a 13% lower mean blood glucose level (26.1 ± 6.1 mmol/L; *P* < 0.001 vs [D]) (Fig 2A). The modest impact of linagliptin on glycemia is reflected by the lack of differences in body weight between the two diabetic groups (Fig 2B). The differences in glycemia were reflected by changes in HbA_1c_ levels. HbA_1c_ was elevated 2.5-fold in diabetic animals, compared with non-diabetic controls ([N] 5.7 ± 0.6% vs [D] 14.2 ± 2.2%; *P* < 0.001). Linagliptin reduced HbA_1c_ by only 14% (to 12.2 ± 1.9% vs [D]; *P* < 0.001) (Fig 2C). We also asked whether linagliptin treatment resulted in reduced levels of reactive metabolites. Under diabetic conditions, we found a 3.5-fold increase in free MG ([N] 1.1 ± 0.2 nmol/mg protein vs [D] 3.8 ± 0.4 nmol/mg protein; *P* < 0.001) and that linagliptin treatment reduced MG concentration by 66% (to 2.0 ± 0.4 nmol/mg protein vs [D]; *P* < 0.001) (Fig 2D).

**Fig 3:**
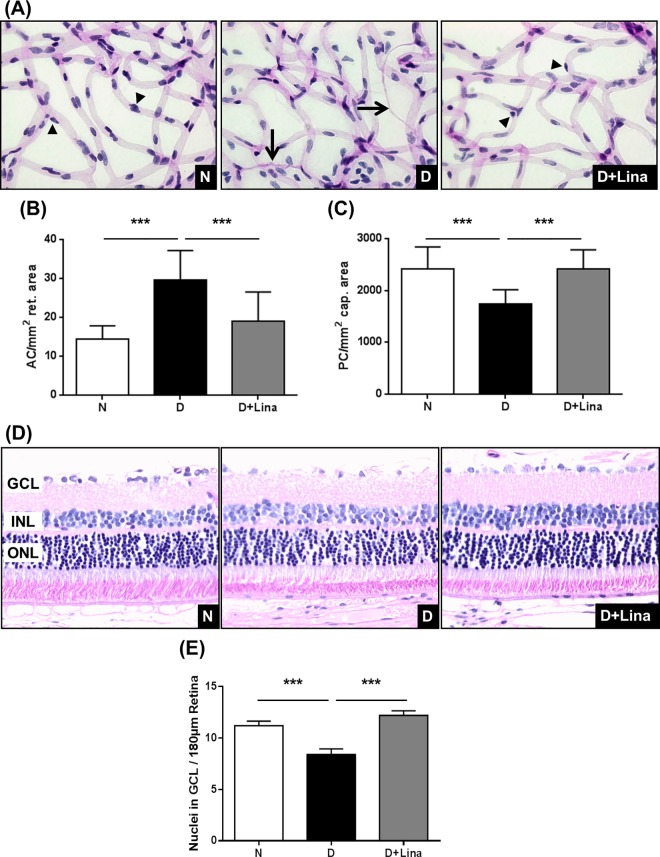
Effect of linagliptin on experimental diabetic retinopathy. (A) Representative images of PAS-stained retinal digest preparations. Arrowheads indicate pericytes [P] and arrows indicate acellular capillaries [AC]. (B) Numbers of acellular capillaries, and (C) pericyte numbers in capillary areas were determined by quantitative retinal morphometry (n = 7). (D) Representative images of PAS-stained paraffin sections (3 μm), (E) numbers of cell nuclei in the ganglion cell layer (GCL) (n = 5). Data are expressed as mean ± SD. **P < 0.01, ***P < 0.001.

The increase in active GLP-1 and lowering of free MG were accompanied by a major reduction in retinal capillary vasoregression. Consistent with previous data, diabetes caused a 2-fold increase in acellular capillaries after 24 weeks of diabetes ([N] 14 ± 3 AC/mm² retinal area vs [D] 30 ± 8 AC/mm² retinal area; *P* < 0.001). Linagliptin reduced acellular capillaries by 70% (19 ± 8 AC/mm² retinal area; *P* < 0.001) (Fig 3A and 3B). Numbers of pericytes were determined using quantitative retinal morphometry of digest preparations. Pericyte numbers were reduced by 28% in diabetic rat retinae (1742 ± 271 PC/mm² retinal area), compared with non-diabetic controls (2416 ± 422 PC/mm² retinal area vs [D]; *P* < 0.001). Consistently, linagliptin reversed pericyte numbers to the levels of non-diabetic animals (2421 ± 363 PC/mm² retinal area vs [D]; *P* < 0.001) (Fig 3A and 3C).

We also assessed possible neuroprotective effects of linagliptin treatment, since DPP4 expression was detected in neurons (Figure in S1 Fig).

In the diabetic group, neuronal cells in the ganglion cell layer (GCL) were reduced by 24% ([N] 11 ± 4 nuclei/180μm retina vs [D] 8 ± 3 nuclei/180μm retina; *P* < 0.001). Indeed, linagliptin treatment reversed cell numbers to normal levels (12 ± 2 nuclei/180μm retina vs [D]; *P* < 0.001) (Fig 3D and 3E).

**Fig 4:**
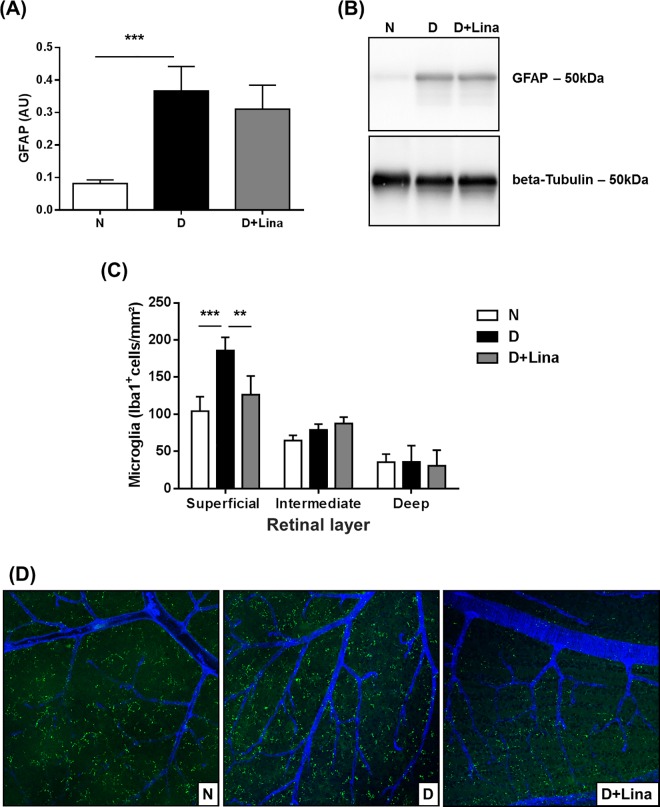
Quantification of glial and microglial activation. (A) Glial activation was quantified by Western blotting using glial fibrillary acidic protein (GFAP) as a marker (n = 5). (B) Representative blot for GFAP and β-Tubulin as loading control.(C) Assessment of microglial activation by quantification of ionized calcium-binding adapter molecule 1 (Iba-1) positive cells (n = 5), and (D) representative immunohistochemistry of the superficial layer in whole mount preparations. Data are expressed as mean ± SD. **P < 0.01, ***P < 0.001.

To study alleviation of diabetic gliosis by linagliptin, GFAP was used as a marker. Its expression was 4.5-fold higher in diabetic rats, compared to normal controls ([N] 0.08 ± 0.01 AU vs [D] 0.4 ± 0.08 AU; *P* < 0.001). However, linagliptin did not prevent diabetic glial activation (Fig 4A and 4B). Moreover, since MG levels are affected by linagliptin treatment and MG activates microglia in experimental diabetic retinopathy [25], we assessed microglial activation by Iba-1 staining. Under hyperglycemia, we observed 78% more Iba-1 positive microglia in the superficial layer ([N] 104 ± 34 cells/mm² vs [D] 186 ± 18 cells/mm²; *P* < 0.001). Linagliptin treatment reduced this increase in Iba-1 positive microglial cells by 73% to almost normal status (126 ± 25 cell/mm²; *P* < 0.01). The intermediate and deep capillary layers were unaffected by diabetes or by linagliptin (Fig 4C and 4D).

To elucidate mechanisms mediating the beneficial effect of linagliptin, expressions of genes with an impact on retinopathy were analyzed, including the pro-inflammatory factors interleukin 1 beta (Il1b) [26] and intercellular adhesion molecule 1 (Icam1) [27], the pro-angiogenic SDF-1a receptor C-X-C motif chemokine receptor 4 (Cxcr4) [28], the antioxidative catalase (Cat) [29], and the vaso- and neuroprotective heme oxygenase 1 (Ho-1) [30].

**Fig 5:**
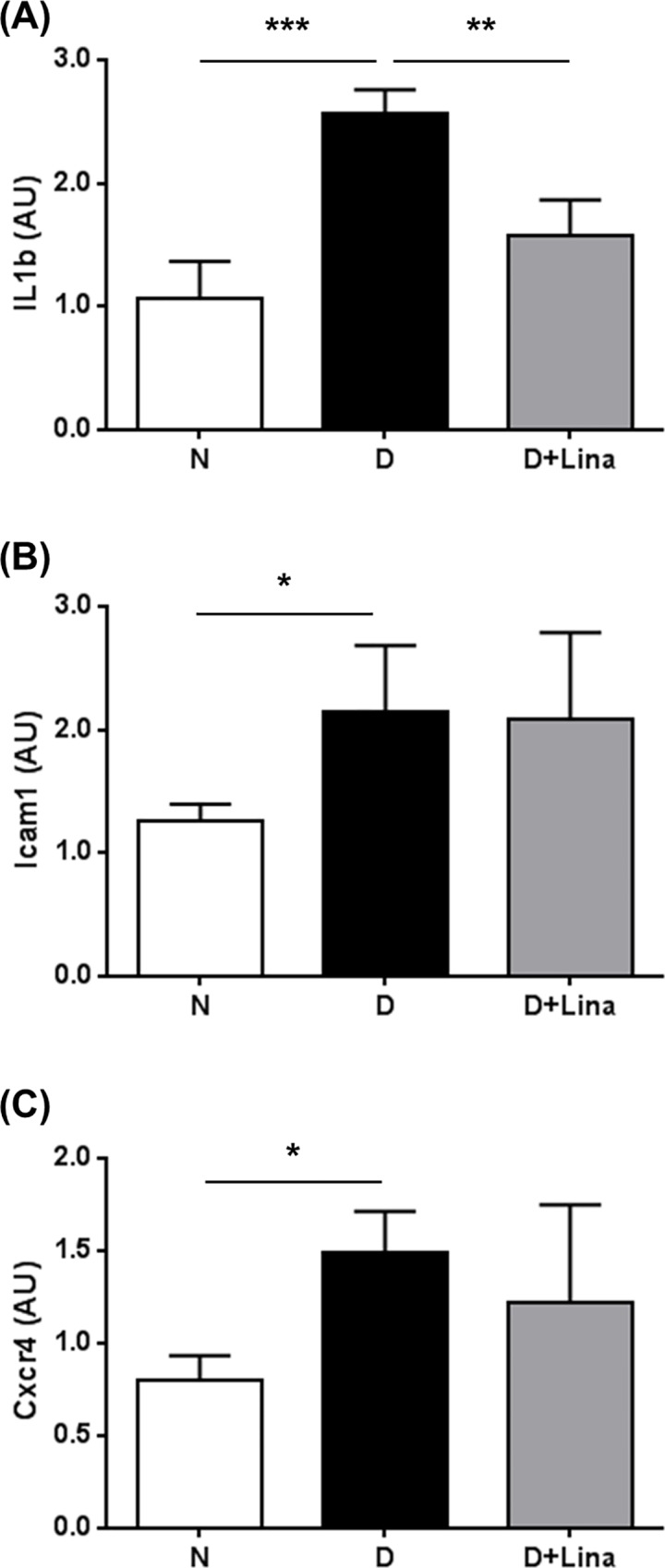
Assessment of inflammatory and angiogenic genes. Expression of pro-inflammatory, (A) Il1b and (B) Icam1, and pro-angiogenic, (C) Cxcr4, markers were determined using by quantitative RT-PCR. Data are expressed as mean ± SD. *P < 0.05, **P < 0.01, ***P < 0.001, (n = 7 for all parameters).

Il1b transcription was upregulated 2.4-fold in diabetic retinae ([N] 1.1 ± 0.3 AU vs [D] 2.6 ± 0.2 AU; *P* < 0.001), and this increase was significantly reduced with linagliptin treatment by 65% (1.6 ± 0.3 AU vs [D]; *P* < 0.01) (Fig 5A). The expressions of Icam1 and Cxcr4 increased 1.7-fold ([N] 1.3 ± 0.1 AU vs [D] 2.2 ± 0.5 AU; *P* < 0.05) and 1.9-fold ([N] 0.8 ± 0.1 AU vs [D] 1.5 ± 0.2 AU; *P* < 0.05), respectively, but did not change with linagliptin (Fig 5B and 5C).

Cat expression was reduced by 20% in diabetic animals ([N] 1.1 ± 0.2 AU vs [D] 0.9 ± 0.04 AU; *P* < 0.05), but was not affected by linagliptin (Figure A in S2 Fig). Ho-1 expression was unaltered by both diabetes and linagliptin treatment (Figure B in S2 Fig).

These data indicate, that linagliptin partially suppresses an Il1b-mediated inflammatory response, involved in apoptosis of retinal capillary cells during development of diabetic retinopathy [31].

In contrast to a previous report [32] but in line with tissue profiling studies of G protein-coupled receptor expression [33], we could not detect GLP-1 receptor expression in the retina, either by quantitative RT-PCR or immunohistochemistry (data not shown). Therefore *C*. *elegans*, a model expressing the orthologue of DPP4 but lacking GLP-1, was used to evaluate GLP-1 independent effects of linagliptin. In previous studies, we demonstrated that high glucose increases ROS formation and linagliptin co-incubation decreases ROS formation by 78%. In addition, we determined the level of MG-derived AGEs to test whether lowering of ROS reduces reactive metabolites. Formation of MG-derived AGEs increased under high glucose and treatment with linagliptin normalized AGE formation almost completely [34].

By assessing body bending frequency as a measurement of neuronal function we could confirm our previous observation [34] that the reduction of reactive metabolites results in neuroprotection.

High glucose decreased body bending frequency ([N] 0.17 ± 0.01 Hz vs [D] 0.14 ± 0.01 Hz; *P* < 0.01). Linagliptin increased body bending frequency under high glucose conditions (0.17 ± 0.03 Hz; *P* < 0.05) (Figure in S3 Fig).

These data suggest that linagliptin improves neuronal function and reduces oxidative stress and AGEs.

## Discussion

In this study we show that the DPP4 inhibitor linagliptin has beneficial effects on several components of the neurovascular unit in the diabetic retina. We observed a substantial rise in circulating levels of active GLP-1, a major reduction of retinal MG, a reduction of retinal vasoregression and microglial activation, and a functional and structural improvement of neuronal cells in two independent model systems. No induction of proliferative retinopathy was observed. We conclude, that linagliptin treatment is favorable for the microvasculature of the diabetic retina through reducing oxidative stress and thus lowering formation of MG and MG-derived AGEs; and through improvement of neuronal function and survival.

Methylglyoxal-derived AGEs are involved in the pathogenesis of diabetic microvascular damage in multiple ways [23, 35]. Although linagliptin reduced glycemia, this moderate GLP-1 mediated effect is unlikely to account for the massive reduction of MG. Importantly, linagliptin is also capable of reducing oxidative stress, indirectly by increasing GLP-1 levels and directly due to its antioxidant properties. Linagliptin is a potent antioxidant according to its 1-electron-oxidations, tested in the hydrogen peroxide/peroxidase system [36]. Indeed, a short-term reduction of oxidative stress in the diabetic retina by linagliptin was reported recently [37]. The antioxidative properties of linagliptin could also explain the systemic nephroprotective effects of DPP4 inhibitors [38].

According to the unifying hypothesis, elevated levels of glucose result in an increased flux through glycolysis and mitochondrial ROS overproduction. This in turn leads to the activation of various biochemical pathways including increased formation of MG and MG-derived AGEs [39]. Direct pharmacological inhibition of MG-derived AGEs or metabolic signal blockers which divert glycolytic intermediates into non-toxic pathways prevents experimental retinopathy [40]. The reduction of MG with linagliptin in our long-term experiment has not been reported previously and represents a mechanism by which exposure to linagliptin may attenuate diabetic microvascular damage. Reduction in MG levels could also explain reduced microglial activation [25].

Normalizing mitochondrial superoxide production blocks pathways of glucotoxic damage [16]. Recently, GLP-1 degradation products such as GLP-1(9–37) and GLP-1(9–36) amide have been described as inhibitors of mitochondrial ROS overproduction [17]. Our experimental setup, increasing active GLP-1 by DPP4 inhibition, is incompatible with the assumption that the antioxidative properties of GLP-1 degradation products prevail the biological effect of active GLP-1, which *per se* lower oxidative stress and AGEs. Linagliptin by itself may be capable of lowering MG generation. The mechanism by which linagliptin lowers MG remains unclear. However, the strong antioxidative capabilities of linagliptin, residing in its xanthine-based molecular structure, may lower the inhibitory effect of ROS on glyceraldehyde 3-phosphate dehydrogenase (GAPDH), thereby reducing the flux of glyceraldehyde 3-phosphate (GA3P) into the AGE pathway. In addition, these results refer to the neurovascular unit whereas the elegant studies conducted by Giacco *et al*. [17] focused on the endothelium of large vessels.

Induction of inflammatory cytokines, either directly through the AGE/RAGE/NFkB axis, or indirectly by interaction of activated white blood cells and the endothelium, contributes to vascular damage in the diabetic retina [4, 41]. Expression of Il1b is upregulated in endothelial cells exposed to high glucose *in vitro* and in the diabetic retina [42]. In our model, Il1b gene expression was consistently upregulated by diabetes. Agents with antioxidant properties such as curcumin and green tea have been shown to reduce both interleukin expression and retinal damage [43, 44]. In contrast, exposure to MG induces the transcription and translation of Il1b, as demonstrated in neuronal cells [45]. With linagliptin treatment we observed significantly reduced Il1b gene expression, presumably due to the combined effect of linagliptin on the levels of both oxidative stress and MG.

Due to an NFkB dependent process whereby pro-Il1b is processed by the inflammasome complex into active Il1b, levels of the active form of the protein cannot be reliably inferred by measuring levels of Il1b mRNA. Therefore, the data presented here related to the role of Il1b must be interpreted with caution. Similarly, linagliptin has no effect on other inflammatory molecules such as Icam1 and Cxcr4 in the retinae of diabetic rats.

Importantly, Il1b induces apoptosis of retinal capillary cells in an NFkB dependent fashion and diabetic Il1b receptor knockout mice are protected from retinal vasoregression [46]. There is substantial evidence, that vasoregression is the most important lesion in preclinical models of diabetic retinopathy and that the reduction of MG-derived AGEs confers protection [40]. However, preclinical models cannot demonstrate an effect of linagliptin on vasoregression, presumably due to insufficient study durations [37]. In the present study, we demonstrate for the first time a protection against diabetic microvascular damage by long-term linagliptin treatment.

Theoretically, linagliptin could also have adverse effects on the neurovascular unit, since DPP4 inhibition may promote the development of proliferative retinopathy. DPP4 is important in the inactivation of proangiogenic factors such as SDF-1a and HMGB-1. The recruitment of bone marrow-derived cells to hypoxic areas, to promote vessel formation in these tissues is supported by SDF-1a. Damaged cells release HMGB-1 and this prepares the microenvironment for repair or inflammatory tissue remodeling. However, the upregulation of SDF-1a and HMGB-1 by hyperglycemia did not reach sufficient levels to induce angiogenesis, since the retina of a STZ diabetic rat does not develop proliferative retinopathy. Additional treatment with linagliptin did not further increase SDF-1a or HMGB-1 and did not result in increased numbers of endothelial cells (EC) (Figure A-C in S4 Fig). The protective effects of linagliptin on the neurovascular unit are not restricted to the vasculature, it also extends to the neuronal compartment. It is known that neuroprotection contributes to vasoprotection in the diabetic retina as demonstrated in animal models [47, 48].

Indeed, our data demonstrated a protective effect of linagliptin on neuronal cell survival and function in two independent model systems. In Wistar rats, exposure to linagliptin conferred complete survival of neuronal cells in the ganglion cell layer under diabetic conditions. Likewise, linagliptin treatment preserved neuronal function in *C*. *elegans*. This neuroprotective effect probably results from direct reduction of ROS and MG, since this animal model harbors DPP4 (*dpf-1* and *dpf-2*) and GLP-1R (*seb-3*) but lacks an ortholog of GLP-1.

Previous work in *C*. *elegans* demonstrated, that modulation of MG levels by gain/loss-of-function of glyoxalase-1 altered different parameters of neuronal function (relative head motility, body bending frequency and body angular velocity), which are all corrected when increased MG was returned to normal levels [23]. In addition, a neuroprotective effect of GLP-1 agonists based on the reduction of ROS was also demonstrated recently in neuroblastoma spinal cord-19 (NSC-19) cells [49].

Recently, GLP-1 receptor (GLP-1R) expression was described in the human retina, suggesting that GLP-1 mediated prevention of retinal neurodegeneration may also involve receptor activation [32]. This is in contrast to both a previous extensive study on tissue profiling of GLP-1R expression [33] and our own data, where expression could not be confirmed. By using established protocols, neither transcription nor translation of GLP-1R could be detected by quantitative RT-PCR and immunohistochemistry, respectively. The use of tissue from rodent sources in the latter studies could be a possible explanation for this discrepancy.

In summary, we found therapeutic properties of linagliptin for the prevention of diabetic vasoregression. Both MG reduction and neuroprotection likely contribute to this effect. There were no signs of proliferative retinopathy upon treatment. Since these changes occur in the absence of major reductions in blood glucose and were also present in GLP-1 receptor-deficient *C*. *elegans*, the vasculoprotective effects of linagliptin are likely independent of glucose and GLP-1 signaling and may instead depend on antioxidative properties. Therapeutic strategies reducing oxidative stress (e.g. dexlipotam, pyridoxamine) and resulting MG (e.g. aminoguanidine, benfotiamine) may confer neuroprotective and ultimately vasculoprotective effects on the neurovascular unit of the diabetic retina. Additional trials, in particular in humans, are required to confirm these observations.

## Supporting Information

S1 Fig Immunohistochemistry of DPP4 in retinal sections. (PDF)

S2 Fig Expression of retinopathy associated factors. (PDF)

S3 Fig Effect of high glucose and linagliptin on neuronal function. Body bending frequency was determined by video analyses. Data is expressed as mean ± SD; *P < 0.05, **P < 0.01.
(PDF)

S4 Fig Effect on proangiogenic factors and vessel formation. (PDF)
