# Supplementary figures and images for: The DPP4 Inhibitor Linagliptin Protects from Experimental Diabetic Retinopathy
Source: PLoS One. 2016 Dec 12;11(12):e0167853. doi: 10.1371/journal.pone.0167853 (PMC5152931; doi:10.1371/journal.pone.0167853)

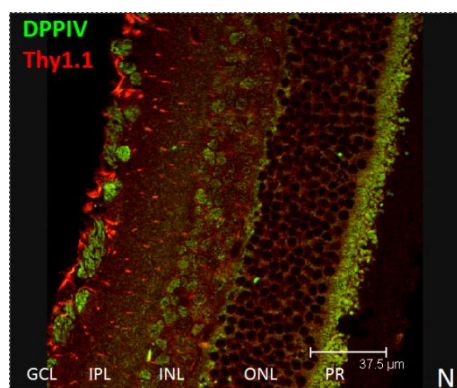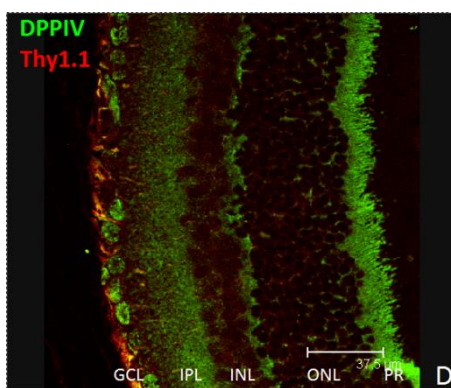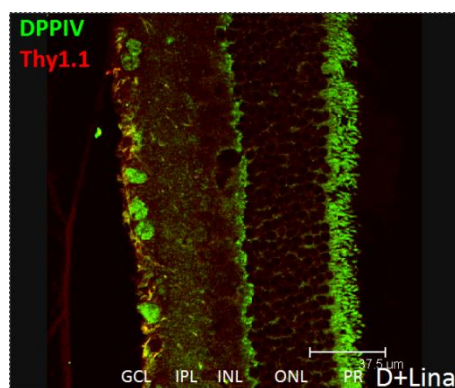

Supplement: S1 Fig — (PDF) [file pone.0167853.s001.pdf]

**(A)**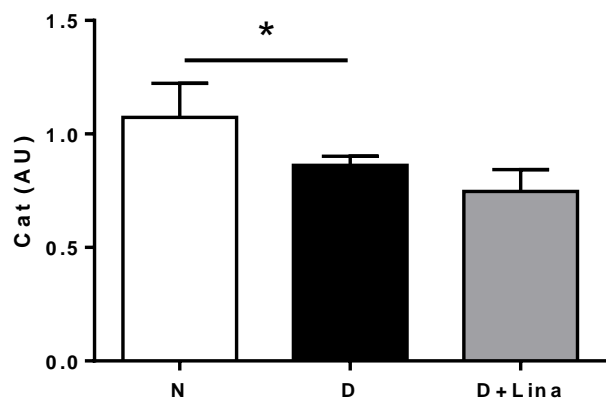**(B)**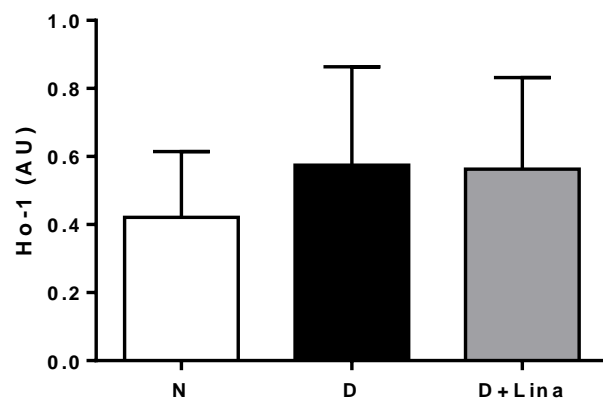

Supplement: S2 Fig — (PDF) [file pone.0167853.s002.pdf]

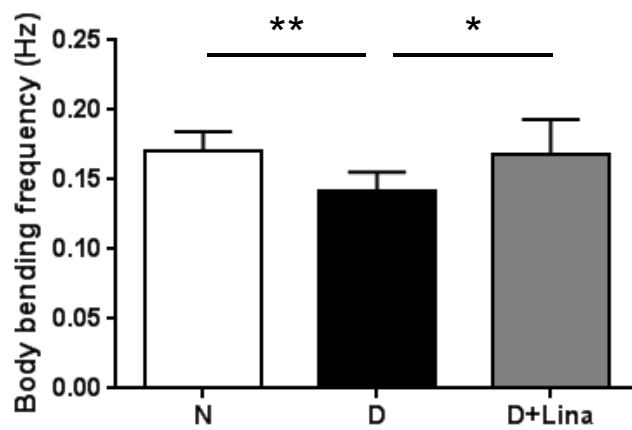

Supplement: S3 Fig — Body bending frequency was determined by video analyses. Data is expressed as mean ± SD; *P < 0.05, **P < 0.01. (PDF) [file pone.0167853.s003.pdf]

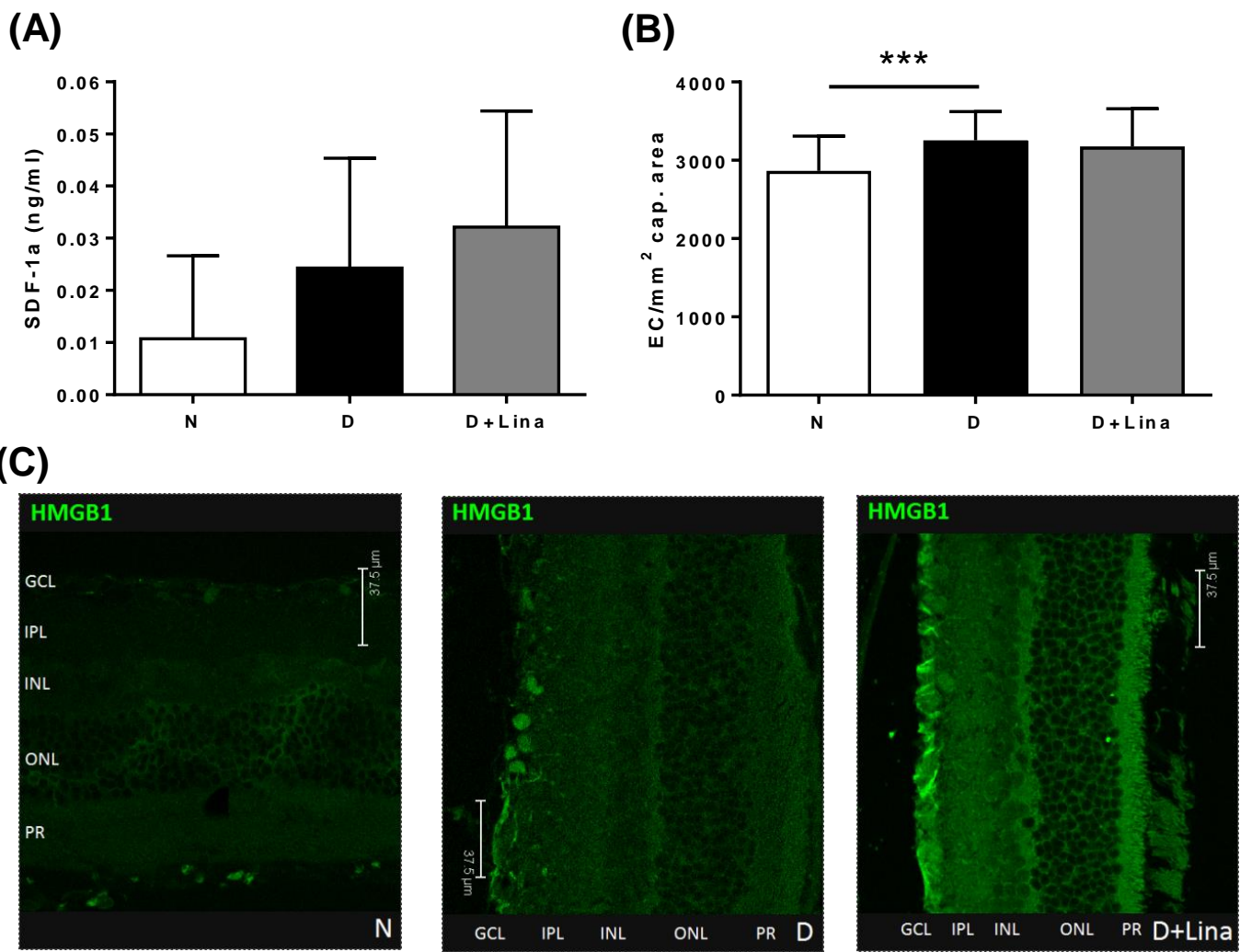

S4 Fig.

Supplement: S4 Fig — (PDF) [file pone.0167853.s004.pdf]
